# Reply to “Generic Statins and Angiotensin Receptor Blockers: Are They Really Useful in Ebola?”

**DOI:** 10.1128/mBio.00094-16

**Published:** 2016-02-23

**Authors:** David S. Fedson, Jeffrey R. Jacobson, Ole Martin Rordam, Steven M. Opal

**Affiliations:** a Retired Physician, Sergy Haut, France; b University of Illinois College of Medicine, Chicago, Illinois, USA; c Practicing Physician, Trondheim, Norway; d Alpert Medical School of Brown University, Providence, Rhode Island, USA

## REPLY

Wikwanikit has several concerns about our report on the effectiveness of treating the host response of patients with Ebola virus disease using a combination of a statin (atorvastatin) and an angiotensin receptor blocker (ARB) (irbesartan) (1, 26). First, not all of the endothelial damage in Ebola described by Baskerville et al. in 1985 was caused by thrombotic events. The ultrastructural changes “took the form of separation of junctions between endothelial cells and detachment from basement membranes. These focal lesions were associated with edema and hemorrhage. Diffuse endothelial injury and loss of endothelial integrity combined with the histochemical changes observed in the animals probably led to hypovolemic shock” (2). A decade later, Feldmann et al. showed that filovirus replication in human monocyte/macrophage cell cultures released inflammatory cytokines that caused an increase in endothelial permeability (3). Virus infection of endothelial cells alone was thought to be insufficient to cause this change (2, 3).

Second, Wiwanikit cites a study that showed higher plasma levels of nitric oxide (NO) in Ebola patients who died than in those who survived (4). High NO levels could have been produced by cytokine-induced overexpression of inducible NO synthase (iNOS) by mononuclear or other cells, or they could have led to the formation of peroxynitrite and other toxic molecules. The balance between iNOS and endothelial NOS (eNOS) is important because eNOS maintains endothelial barrier integrity (5, 6). Wiwanikit also notes that the addition of an NO donor can compromise the antihypertensive effect of ARB treatment (7), but this does not seem relevant to the effects of ARBs on endothelial barrier integrity.

Third, Wiwanikit notes that statin pretreatment failed to attenuate the reduction in forearm-mediated dilatation (FMD) caused by a short episode of ischemia/reperfusion (IR) (8). It is unclear how these changes in healthy subjects correlate with the broader aspects of IR-induced endothelial dysfunction seen in patients with inflammatory diseases such as acute myocardial infarction. In these patients, the anti-inflammatory effects of statins reduce periprocedural major cardiovascular events in patients undergoing percutaneous interventions (9, 10) and in those undergoing noncardiac surgery (11).

Fourth, Wikwanikit suggests that statin treatment of Ebola patients might cause autoimmune-mediated necrotizing myopathy (12). This condition has not been reported in large-scale clinical trials (13), and it occurs in only 2 cases per million years of treatment (14). Moreover, large randomized controlled trials have documented the safety of statin treatment in patients with acute critical illness (see, for example, reference 15). These reports provide reassurance on the safety of treating Ebola patients with atorvastatin and irbesartan.

Ebola scientists have been reluctant to consider treatments that target the host response (16). Instead, they favor targeting the virus with agents shown to be promising in nonhuman primate models of Ebola. In September 2014, the World Health Organization prioritized several of these antiviral agents and convalescent plasma for clinical trials in West Africa (17). One antiviral agent (favipiravir) was shown to reduce mortality in patients with low virus loads, but it failed to affect the 85% mortality in those with high virus loads (18). None of the other trials was successful, leading two observers to call the overall Ebola clinical trial experience a “thin scientific harvest” (19).

Atorvastatin and irbesartan have broad anti-inflammatory effects (20, 21), and combination treatment is more effective than treatment with either agent alone (22). Despite the opposition of Ebola scientists (16), we assumed that treatment with these drugs would maintain or restore endothelial barrier integrity (23), an assumption that has strong biological plausibility (24). Despite the reservations of Wiwanikit, the experience of physicians in Sierra Leone indicates that treating the host response in Ebola patients substantially improved survival (1, 25).
